# Structural evolution and stabilities of (CuIn)_*n*_Te_2_ and ((CuIn)_*n*_Te_2_)^−^ (*n* = 1–8) clusters *via* DFT study

**DOI:** 10.1039/d6ra00246c

**Published:** 2026-01-28

**Authors:** Kidane Goitom Gerezgiher, Bereket Woldegbreal Taklu, Taame Abraha Berhe, Teklay Mezgebe Hagos, Hagos Woldeghebriel Zeweldi

**Affiliations:** a Department of Physics, Abbiyi Addi College of Teachers Education and Educational Leadership Abbiyi Addi, P. O. Box 11 Ethiopia kidanegoitom5@gmail.com; b Department of Chemistry, CNCS, Wollo University P. O. Box 1145, Dessie Ethiopia; c Department of Chemistry, CNCS, Adigrat University P. O. Box 50 Adigrat Ethiopia; d Department of Physics, CNCS, Mekelle University P. O. Box 231 Mekelle Ethiopia hagos93@mu.edu.et

## Abstract

CuInTe_2_ is a promising semiconductor with a tunable bandgap of 1.0-1.2 eV, enabling it to efficiently absorb sunlight and convert it into usable energy. Following this development, characterization of its structural and electronic properties is currently underway. In this study, the Vienna *Ab Initio* Simulation Package (VASP) with density functional theory (DFT) and plane-wave basis sets was used to investigate the structural and electronic properties of both neutral and anionic clusters. For (CuIn)_*n*_Te_2_ and ((CuIn)_*n*_Te_2_)^−^ (*n* = 1–8) clusters, geometric optimization revealed the lowest-energy isomers, all of which adopt cubic chalcopyrite structures. According to the results, the low-lying energy geometry of Cu_2_In_2_Te_2_ and (CuInTe_2_)^−^ clusters exhibit their maximum relative stability. The (CuIn)_*n*_Te_2_ thin-film experimental finding of 1.85 eV is a good match with their mean HOMO–LUMO gaps of 1.652 eV and 2.464 eV. Binding energy per atom increases with cluster size, although the HOMO–LUMO gap breaks at *n* = 5, most likely as a result of bond-specific interactions and orbital hybridization. The Cu_2_In_2_Te_2_ cluster stands out with maximum HOMO–LUMO gap and dissociation energy, consistent with its enhanced stability. Adiabatic ionization potentials decrease with cluster size, indicating growing metallic character, while dissociation energies show odd–even oscillations but overall increase as size grows. Partial charge density analysis shows that both neutral and anion clusters are significant for semiconductor applications, including photovoltaic cells and related devices.

## Abstract 2

CuInTe_2_ is a promising semiconductor with a tunable bandgap between 1.0 and 1.2 eV, allowing it to efficiently absorb sunlight and convert it into usable energy.

## Graphical abstract

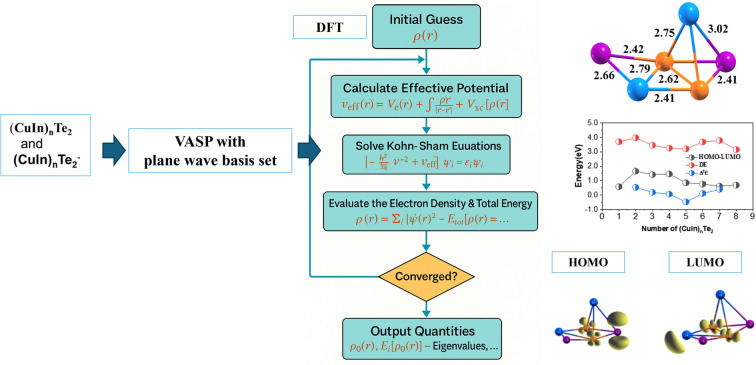

## 1 Introduction

Transition-metal–semiconductor nanoclusters, particularly chalcopyrite (group I, III, and VI elements), have long been of interest to physicists and chemists due to their potential as constituent elements of novel nanomaterials.^1–6^ Coin metals form ternary chalcopyrite I–III–VI_2_ compounds with semiconductors such as germanium, indium, arsenic, selenium, gallium, sulfur, and tellurium, and are crucial for the cost-effective production of thin-film solar cells using various experimental techniques.^7^

The electronic and structural properties of clusters combining zinc, silver, gold, and copper with semiconductors have been investigated in a series of experimental and theoretical studies. However, challenges remain in achieving precise crystallization of these nanomaterials. In this regard, pyrolysis and electrodeposition are viable experimental methods to fabricate thin-film solar cells like CuInTe_2_.^8^ Semiconductors like ternary chalcopyrite I–III–VI are promising current research interests while numerous materials are suitable for converting solar energy *via* photovoltaic processes.^9,10^ CuInTe_2_ belongs to the I–III–VI ternary chalcopyrite semiconductor compound family and is of paramount importance for electro-optical devices, integrated optics, solar cells, optoelectronics, and thermoelectric, applications.^11–13^

As reported by Adhikari S. and N. Chaure *et al.*,^14^ the energy bandgap, structural characteristics, surface morphology, and elemental composition of (CuIn)_*n*_Te_2_ thin films were elucidated in.^15^ The obtained thin films exhibited polycrystalline behavior, with bandgaps ranging from 1.27 to 1.89 eV. Yarema *et al.*^16,17^ investigated composition tuning of multicomponent semiconductor nanocrystals, an example of I–III–VI materials, reporting that stoichiometric CuInSe_2_ is a direct bandgap semiconductor with a bandgap of 1.04 eV.^18^ Numerous theoretical and experimental efforts have been undertaken to elucidate the structure and properties of small scale (CuIn)_*n*_Te_2_ and related clusters. CuInTe_2_, belongs to the I–III–VI_2_ ternary chalcopyrite semiconductor compound family and is of paramount importance for applications in thermoelectric, optoelectronics, solar cells, integrated optics, and electro-optical devices.

Furthermore, it has been reported that Cu–In–Se phases such as CuIn_3_Te_5_ and CuIn_5_Te_8_ exhibit larger band gaps, direct type, 1.21 eV and 1.15 eV, respectively. In this study, we investigated the electronic and structural properties of (CuIn)_*n*_Te_2_ in neutral and anionic cluster forms using density functional theory. Our findings reveal that cluster size and charge state influence the electronic properties and structural stability of (CuIn)_*n*_Te_2_. Consequently, Cu_2_In_2_Te_2_/(Cu_2_In_2_Te_2_)^−^ was found to exhibit excellent stability in both neutral and anionic forms, owing to favorable electronic interactions and bonding chemistry.

## 2 Computational method

The VASP is used to examine the electronic and structural properties of neutral and anionic, (CuIn)_*n*_Te_2_ clusters.^19^ The package uses density functional theory (DFT) with a plane-wave and ultrasoft pseudopotential basis set. The arrangement of atoms inside a supercell that repeats on a regular basis serves as the input for VASP. Atomic mobility and the electrical structure and energy of the atomic configuration are the most basic outputs. If the cluster length is less than 10 Å, we employed a cubic supercell with an edge length of 20 Å simulation box for structure optimization; if not, we used a (30 × 30 × 30) Å^3^ simulation box with periodic boundary conditions.^20^ Furthermore, due to the size of the supercell, only the point (*Γ*) is used to scan the Brillouin zone. VASP uses a line minimization of the energy along the force direction to determine the minimum. The atoms are relaxed to their present ground state using a conjugate gradient method. Band structure energy is integrated across the entire Brillouin zone using the smearing or tetrahedron method. The density of states was determined using a Gaussian smearing of 0.01 eV. For each system, the plane-wave cutoff energy was set to 240 eV.^20^ The structure was considered to have converged when the force acting on each ion, as calculated using the Kohn–Sham energy function, was less than 10^−4^ eV Å^−1^. Quantification of various planar and three-dimensional structures was performed using the conjugate gradient method. The atomic electron configurations and number of electrons considered for each parent molecule were (3d^10^4s^1^) for copper (Cu), (5s^2^5p^1^) for indium (In) and (5s^2^5p^4^) for tellurium (Te). In order to ascertain whether the lowest-energy structure had been discovered, we placed copper, indium and tellurium atoms in multiple hypothetical arrangements, both symmetrical and non-symmetrical.

## 3 Results and discussion

After optimizing the minimal energy structure, energy parameters such as the second-order energy difference, ionization energy, binding energy, dissociation energy and HOMO–LUMO gap are calculated to confirm the relative stability of the isomers. The amount of energy required at the moment of creation or the amount of energy we need to add to the system to disintegrate it is considered to be the binding energy.^21^ The binding energy formed by the pair (A_*x*_B_*y*_) can be obtained from eqn (1).1
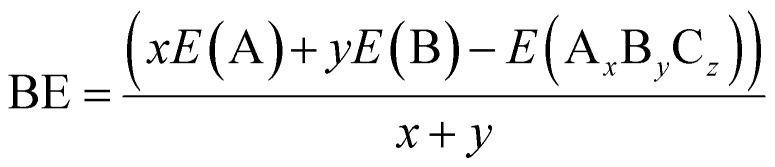


For three atoms cluster, it is explained as2
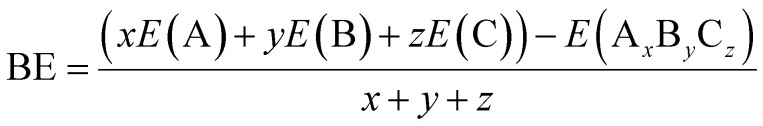
Here, *E*(A), *E*(A_*x*_B_*y*_C_*z*_) denotes the minimum energy of the corresponding cluster, variable such as *x*, *y*, *z* denote the number of individual atoms, and *E*(B), *E*(C) represent the minimum energies of individual atoms A, B, C respectively. Other properties of clusters are dissociation energy and second-order energy difference. The second-order energy difference (Δ^2^*E*_b_) and dissociation energy (DE) are extremely sensitive indicators in the context of cluster physics that accurately reflect the relative stabilities of the systems being studied.^21^ They help us to understand the energetic behavior of the clusters as the given molecule is liberated.^22^ The former is used to calculate the energy needed to remove a particular molecule from the mother cluster. For comparison purposes, dissociation energy (DE) and second-order difference in binding energy are calculated using eqn (3) and (4).

3 DE = *E*(A_*x*−1_B_*y*_) − *E*(A) − *E*(A_*x*_B_*y*_)

4 Δ^2^*E*_b_(A_*x*_B_*y*_) = *E*(A_*x*−1_B_*y*_) + *E*(A_*x*+1_B_*y*_) − 2*E*(A_*x*_B_*y*_)

where, *E* represents the total energy. The energy difference between the LUMO and the HOMO indicates how likely it is that molecular orbitals will participate in chemical reactions, and how likely it is that electrons will be transferred from occupied orbitals to unoccupied ones^5^eqn (5) is used to determine the energy difference between HOMO and LUMO:

5 [HOMO–LUMO]_gap_ = *E*_LUMO_ − *E*_HOMO_

Ionization energy (IP), electron affinity (EA), and dissociation energy (DE) are additional key factors in verifying the stability of clusters. The energy difference between the lowest-energy neutral species with a neutral structure and the cationic structure is known as the vertical ionization potential (VIP). Conversely, the energy difference between the lowest-energy anion and the neutral species with an anionic structure is known as the vertical dissociation energy (VDE).

In contrast, in the AIP and ADE, respectively, the energy difference between the structures with the lowest energy of the neutral particle and the cation, or the neutral particle and the anion, defines the adiabatic case. The energy required to produce a cation in its ground state by removing an electron from the ground state of the neutral system is known as the adiabatic ionization energy.^23^ These values correspond to the enthalpy change at 0 K, but the vertical electron attachment energy and EA are defined identically to the adiabatic ionization energy and vertical ionization energy respectively. These quantities can all be expressed numerically using eqn (6)–(8).

6 AIP = *E*_ground state_^+^ − *E*^0^_ground state_

7 VDE = *E*^0^_geometry of anion_ − *E*_ground state_^−^

8 ADE = *E*^0^_ground state_ − *E*_ground state_^−^

where *E* is the energy, and the superscripts represent the charge of the cluster. If a cluster has low electron detachment energy, the neutral cluster prefers not to accept one unit of charge; however, a large electron detachment energy is an indication that the anion is the more stable species. A large ionization potential indicates that the neutral cluster is more stable than the cation. The following section examines the growth of these clusters in relation to the number of molecular units (CuIn)_*n*_Te_2_ and ((CuIn)_*n*_Te_2_)^−^ (*n* = 1–8).

### 3.1. Dimer

Before dealing with the greater size clusters, we will do basic calculations of the neutral dimers as follows:

#### 3.1.1. Cu–Cu dimer

Beginning with Cu–Cu dimer, the bond lengths of neutral, cation, and anion is found to be 2.15 Å, 2.73 Å, 2.35 Å, respectively, which is in good agreement with the work of ref. 24 and 25. The estimated structural parameters for Cu_2_ and (Cu)_2_^−^ exhibit typical LDA accuracy in bond length calculations and agree well with experimental results. These equations accurately reproduce the increase in bond lengths and the softening of potentials observed during the transition from neutral dimers to anions. The electronic structure of the neutral dimer provides an explanation for the long bond lengths and the tendency towards a relaxed potential observed in both the anionic and cationic dimers.

The neutral dimer is a closed-shell system possessing a gap of 1.629 eV between its highest occupied molecular orbital (HOMO) and lowest unoccupied molecular orbital (LUMO). This substantial gap suggests that the HOMO state exhibits significantly stronger bonding characteristics than the LUMO state. During the transition from a neutral dimer to a cationic dimer, the removal of electrons from the bonding HOMO state leads to an overall weakening of the bond and an increase in bond length. The binding energy of the cation is 5.772 eV, while the HOMO–LUMO gap is 0.01 eV. In the anion dimer, adding an electron to the half-filled LUMO state results in weaker bonds and longer bond lengths. The bond energy and HOMO–LUMO gap were confirmed to be 7.246 eV and 1.535 eV, respectively.

By comparing the total energy of the relaxed neutral, cationic, and anion dimers, we calculate that the adiabatic and vertical detachment energies of the dimer are found to be 0.0361 eV and 1.904 eV, respectively. Similarly (though not illustrated in the figure), the adiabatic ionization energy is 2.912 eV. Removing an electron from the dimer requires more energy than from an isolated Cu atom, indicating a more stable electronic configuration in the dimer. This stability could be due to: bonding (sharing electrons between the Cu atoms leads to a delocalized molecular orbital that's lower in energy than the atomic orbitals, making it harder to remove an electron). Symmetry (electronic configuration of the dimer might have higher symmetry and degeneracy, leading to a more stable ground state). Larger AIP differences suggest stronger covalent character, where electrons are shared significantly between the atoms.

#### 3.1.2. Cu–In dimer

The computational bond lengths are found to be 2.44 Å, 2.43 Å, and 2.43 Å for neutral, cation, and anion Cu–In dimer, which is in good agreement with ref. 11, which is 3.08 Å and the type of bonding between Cu–In is covalent bonding. The binding energies of the neutral, cation, and anion Cu–In dimer are calculated to be 1.621 eV, 12.304 eV, and 28.196 eV, respectively. The HOMO–LUMO gap energy is estimated to be 1.702 eV, 0.202 eV, and 0.055 eV, respectively. The values of AIP, VDE, and ADE are −21.339 eV, 1.780 eV, and 24.927 eV, respectively. The binding energy of the anionic Cu–In dimer is the highest compared to both the cationic Cu–In dimer and the neutral Cu–In dimer. It suggests extra electron stabilization: the presence of an extra electron in the anionic dimer introduces a negative charge, which can attract the positively charged copper and indium ions, leading to stronger electrostatic interactions and a more stable structure. The extra electron might be delocalized across the dimer, leading to charge transfer between Cu and In and more favorable electronic configurations, thereby strengthening covalent bonding. Moreover, anions often have larger radii than neutral atoms, allowing greater relaxation and more favorable packing within the dimer, potentially contributing to increased stability.

#### 3.1.3. Cu–Te dimer

The bond length between Cu–Te of neutral, cationic, and anionic compounds is computed to be 2.30 Å, 2.33 Å, and 2.34 Å, respectively. This matches the findings of ref. 26 and 27 well. The predicted binding energies of the anionic Cu–Te dimer, the cationic Cu–Te dimer, and the neutral Cu–Te dimer are 10.782 eV, 12.588 eV, and 1.937 eV, respectively. Furthermore, the neutral, cationic, and anionic have HOMO–LUMO gap energies of 1.701 eV, 0.039 eV, and 0182 eV, respectively. It is discovered that the AIP, VDE, and ADE are, respectively, −21.302 eV, −0.417 eV, and 17.689 eV.

The binding energy of the cationic Cu–Te dimer is the highest compared to the Cu–Te anionic and neutral dimers, which may be due to increased electrostatic attraction: positive charge on the Cu atom in the cationic dimer enhances the electrostatic attraction towards the Te atom, leading to a stronger overall bond. This is because opposite charges attract, pulling the Cu and Te closer together compared to the neutral or anionic counterparts. The cationic charge might be delocalized across the entire dimer, leading to more favorable orbital interactions between Cu and Te. This can contribute to stronger covalent bonding, further stabilizing the cationic dimer.

Moreover, the different oxidation states of Cu in the three dimers can significantly influence the bonding character. Cationic Cu (+1) tends to form more covalent bonds, while neutral (0) and anionic Cu (−1) have more ionic character. This implies that the cationic Cu–Te dimer benefits from both electrostatic and covalent interactions, resulting in a higher binding energy. Cationic Cu–Te dimer with high binding energy could be interesting candidates for various applications due to their enhanced stability and unique properties. These might include materials with improved conductivity, catalysis, or sensing capabilities.

#### 3.1.4. In–Te dimer

The bond length of In–Te neutral, cationic, and anionic dimers is found to be approximately 2.58 Å, 3.00 Å, and 2.74 Å, respectively. The binding energies of neutral, cationic, and anionic In–Te dimers are calculated to be 1.963 eV, 38.247 eV, and 40.427 eV, respectively. Moreover, the HOMO–LUMO gap energy is estimated to be 2.995 eV, 0.093 eV, and 0.030 eV, respectively. The AIP, VDE, and ADE of In–Te dimer are found to be −72.987 eV, 2.043 eV, and 77.179 eV, respectively, for neutral, cationic, and anionic dimer. The anionic In–Te dimer has the highest binding energy and adiabatic detachment energy compared to the cationic and neutral dimers, indicating a stronger electrostatic attraction between the indium and tellurium atoms in the anionic dimer.^28^ This suggests a more stable and favorable electronic configuration in the anionic state. The high detachment energy indicates that the anionic dimer has a strong affinity for the extra electron, making it more difficult to remove than the other dimers. This could be due to factors like favorable electronic orbital overlap or effective screening of the nuclear charge by the additional electron. Moreover, the combined high binding and detachment energies suggest that the anionic In–Te dimer might be more stable than the cationic and neutral forms, especially in environments where it can retain the extra electron. This could have implications for its potential applications in materials science or anion chemistry. While energetic trends suggest a more stable anionic dimer, it's essential to consider other factors, such as geometry, vibrational frequencies, and solvent effects, to gain a more comprehensive understanding of its stability and behavior.

#### 3.1.5. In–In dimer

As seen from optimized results, the bond length of (In–In), (In–In)^+^ and (In–In)^−^ is longer than the In_2_^+^ and (In–In)^−^, which is 2.97 Å, 2.68 Å, and 2.69 Å. According to ref. 29 the bond length of In–In dimer is found to be 2.97 Å, and the type of bonding between In–In is covalent bonding. The optimized results indicate that the ground-state structures of In_2_^1±^ closely resemble that of the neutral In_2_ cluster, and all three (neutral, cationic, and anionic) share the same symmetry.

The binding energies are 0.819 eV, 40.432 eV, and 42.549 eV, respectively. The HOMO–LUMO gap energy of neutral In–In is calculated to be 1.595 eV, 0.147 eV, and 0.079 eV, respectively. The AIP, VDE, and ADE are found to be −79.206 eV, 2.127 eV, and 83.441 eV, respectively. The binding energy of the anionic In–In dimer is the highest among cationic and neutral In–In dimers. The additional electron in the anionic dimer creates a negative charge, leading to electrostatic attraction between the two indium atoms. This attraction can overcome the repulsive forces between the positively charged nuclei, resulting in a stronger bond and higher binding energy compared to the neutral and cationic dimers. The negative charge might not be localized on a single indium atom but rather delocalized across both atoms. This delocalization can lead to stronger bonds due to increased orbital overlap and resonance stabilization. The extra electron could occupy specific orbitals that interact favorably with the indium atoms' orbitals, leading to stronger covalent bonding than in neutral or cationic dimers.

#### 3.1.6. Te–Te dimer

The average bond length of Te_2_, Te_2_^+^, and Te_2_^−^, is found to be 2.54 Å, 2.81 Å, and 2.82 Å respectively which is in good agreement with the result of ref. 30. The binding energies of the neutral, cationic, and anionic tellurium dimers are calculated to be 2.407 eV, 38.901 eV, and 40.997 eV, respectively, which is in good agreement with ref. 31 and 32. Moreover, the HOMO–LUMO gap energy is 2.653 eV, 0.151 eV, and 0.028 eV, respectively. The AIP, VDE, and ADE are found to be −72.987 eV, 2.043 eV, and 77.179 eV, respectively. The highest binding energy and adiabatic detachment energy indicate a much stronger bond between the two Te atoms in the anionic dimer than in the cationic and neutral dimers.^33^ This strong bond could be due to several factors, such as: ionic character: the anion likely gains an electron, forming a Te^2−^ ion. This negative charge can attract the positively charged Te nucleus, leading to a strong electrostatic attraction. There could be significant charge transfer between the Te atoms, creating a partial positive and negative charge distribution that strengthens the bond. The specific electronic configuration of the anionic dimer might favor a particularly stable bonding arrangement. The high adiabatic detachment energy indicates that removing an electron from the anionic dimer is more difficult than from the other dimers. This suggests that the extra electron is well-stabilized within the anionic structure. Due to the strong bond and stability, the anionic Te–Te dimer could be interesting for various applications, such as: building nanomaterials with unique properties and the strong Te–Te bond and ability to bind other molecules could make the dimer a good candidate for catalytic applications.

### 3.2. Structural evolution of (CuIn)_*n*_Te_2_ and ((CuIn)_*n*_Te_2_)^−^ clusters

#### 3.2.1. CuInTe_2_/(CuInTe_2_)^−^ clusters

**Fig. 1:**
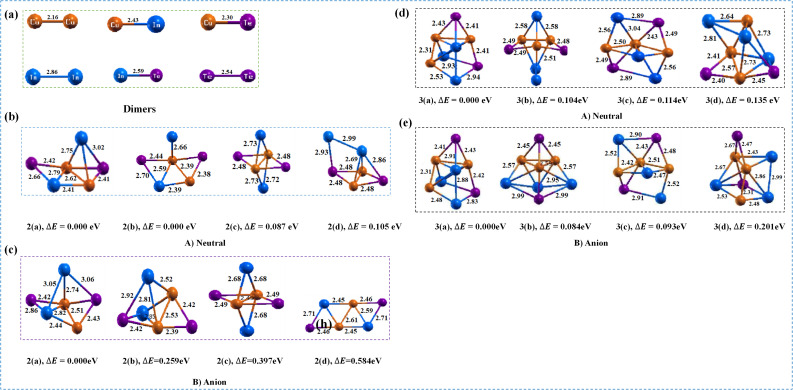
Optimized geometry of dimers (a), (CuIn)_*n*_Te_2_ (b and d), and (CuIn_*n*_Te_2_)^−^ (c and e) clusters for (*n* = 1–3), clusters for (*n* = 1–3), with corresponding relative energy Δ*E*, where the total energy is normalized to the lowest energy.

This study indicate that three distinct and stable structural clusters have been selected for the tetratomic neutral cluster of CuInTe_2_: one 3D and two 2D. The 2D cluster has a Cu–In bond in the center with bond lengths Cu–In, Cu–Te and In–Te of approximately 2.47 Å, 2.51 Å and 2.61 Å, respectively, and appears to be the most stable structure.^5,34^ The most stable of the three configurations is the first isomer in Fig. 1(a), which has a flat rhombic shape. It has an energy preference of 0.129 eV over the second isomer and 0.275 eV over the third isomer. The calculated binding energy and HOMO–LUMO gap for the first isomer are 2.942 eV and 0.577 eV, respectively,^5^ indicating improved stability. The lowest-energy anionic isomer is more stable than the comparable neutral form, and these structures favor distorted configurations compared to their neutral counterparts. Interestingly, the bond lengths of the anionic clusters differ significantly from those of the neutral isomers. The Cu–In, Cu–Te, and In–Te bond lengths for the first isomer (Fig. 1(b)) are 2.55 Å, 2.42 Å, and 2.79 Å, respectively, and differ significantly from the neutral structure.^5^ The flat rhombic, distorted double bridge and flat double bridge structures are represented by the predicted ADEs/VDEs of 3.53/3.78 eV, 4.12/2.58 eV and 2.26/2.48 eV for the three (CuInTe_2_)^−^ isomers.^5^

#### 3.2.2. Cu_2_In_2_Te_2_/(Cu_2_In_2_Te_2_)^−^ clusters

When the CuIn unit is allowed to have a bond with isomer 2(a) of neutral cluster one,^5^ all the possible input configurations are converged to minimum energy structures shown in Fig. 1(b). In general, the 3D structures were found to have lower energy than the 2D structures. As shown in Fig. 1(b), (2a–2d) isomers are considered for neutral Cu_2_In_2_Te_2_ clusters. Isomer 2(a) and isomer 2(b) are degenerate and have different geometries. Isomer 2(c) has 0.087 eV less energy than 2(d) with 0.105 eV low energy. The Cu–Cu bond length is found to be 2.62 Å, measured at the center of the neutral cluster. Cu–In, Cu–Te and In–Te bonds with typical bond lengths of 2.69 Å, 2.42 Å and 2.84 Å are generated therein. The structure of isomer 2(a) is a flat rhombus attached to a triangular prism. The average bond angle of the prism, *i.e.* Cu–In – Te, is 49.2° and the average angle of In–Te–Cu is 62.4°. Isomers 2(a) and 2(b) are energetically degenerate. The HOMO–LUMO gap and binding energy are determined to be 1.652 eV and 3.166 eV, respectively.

**Fig. 2:**
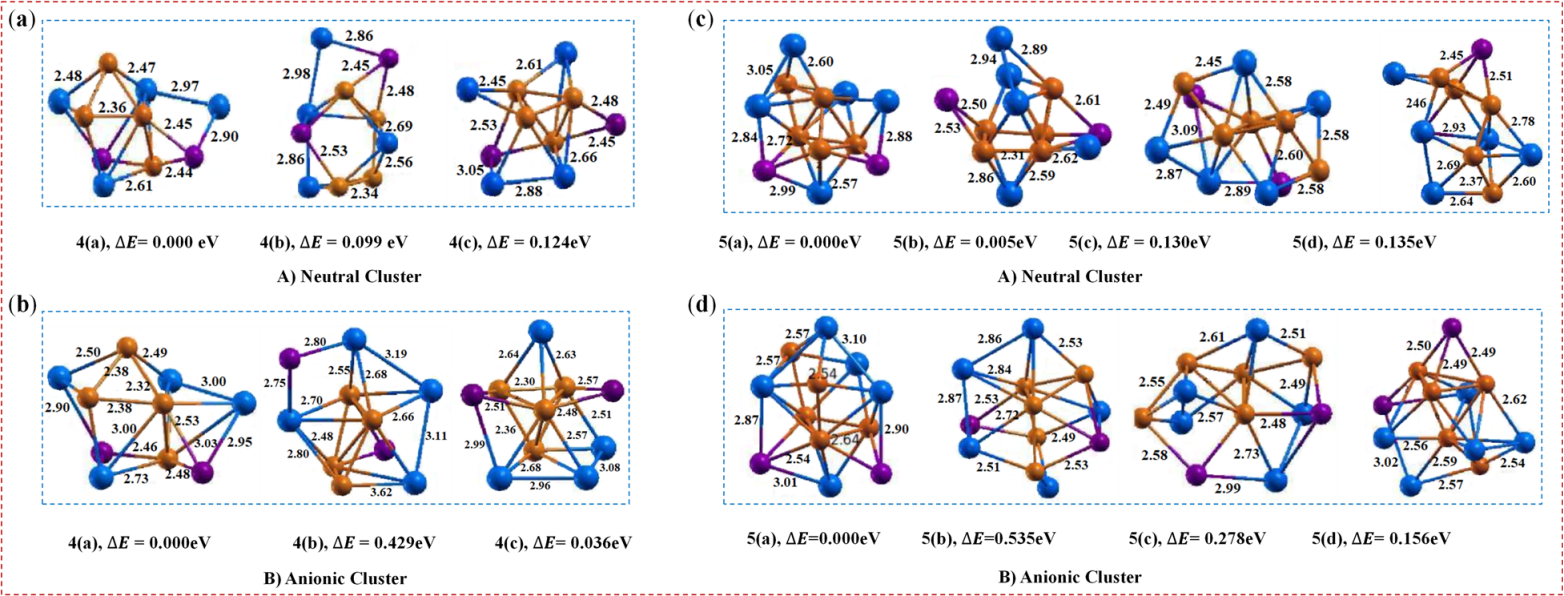
Optimized geometry of (CuIn)_*n*_Te_2_ (a, and c) and ((CuIn)_*n*_Te_2_)^−^ (b, and d) clusters for (*n* = 4–5), with corresponding relative energy Δ*E*, and the total energy is normalized to the lowest energy.

As it is shown (Fig. 1(c), 2(a–d)), the anionic part differs in structure and atom orientation from the neutral structure. Isomer 2(a) has an additional In–In with a bond length of 3.05 Å. Additionally, Cu–Te and Cu–In have bond lengths between 2.39 Å and 2.43 Å and 2.44 Å and 2.82 Å, respectively. Comparing Fig. 1(d and e), isomer 2(c) has the same structure as the neutral structure except in its bond length. The bond length of Cu–Te and Cu–Cu is 2.49 Å. In isomer 2(c), the bond length of Cu–In is found to be 2.68 Å, which decreases by 0.05 Å from the respective neutral. The additional bond of In–In in this anionic cluster is due to the repulsive forces between like charges and the resulting structural adjustments. Moreover, anionic clusters often experience lattice distortion due to the presence of charges, which in turn leads to an increase in bond length compared to neutral clusters, where such repulsive forces are absent or minimized. The ADE and VDE of the low-lying isomer are 2.37 eV and 2.85 eV, respectively.

#### 3.2.3. (CuIn)_3_Te_2_/((CuIn)_3_Te_2_)^−^ clusters

**Fig. 3:**
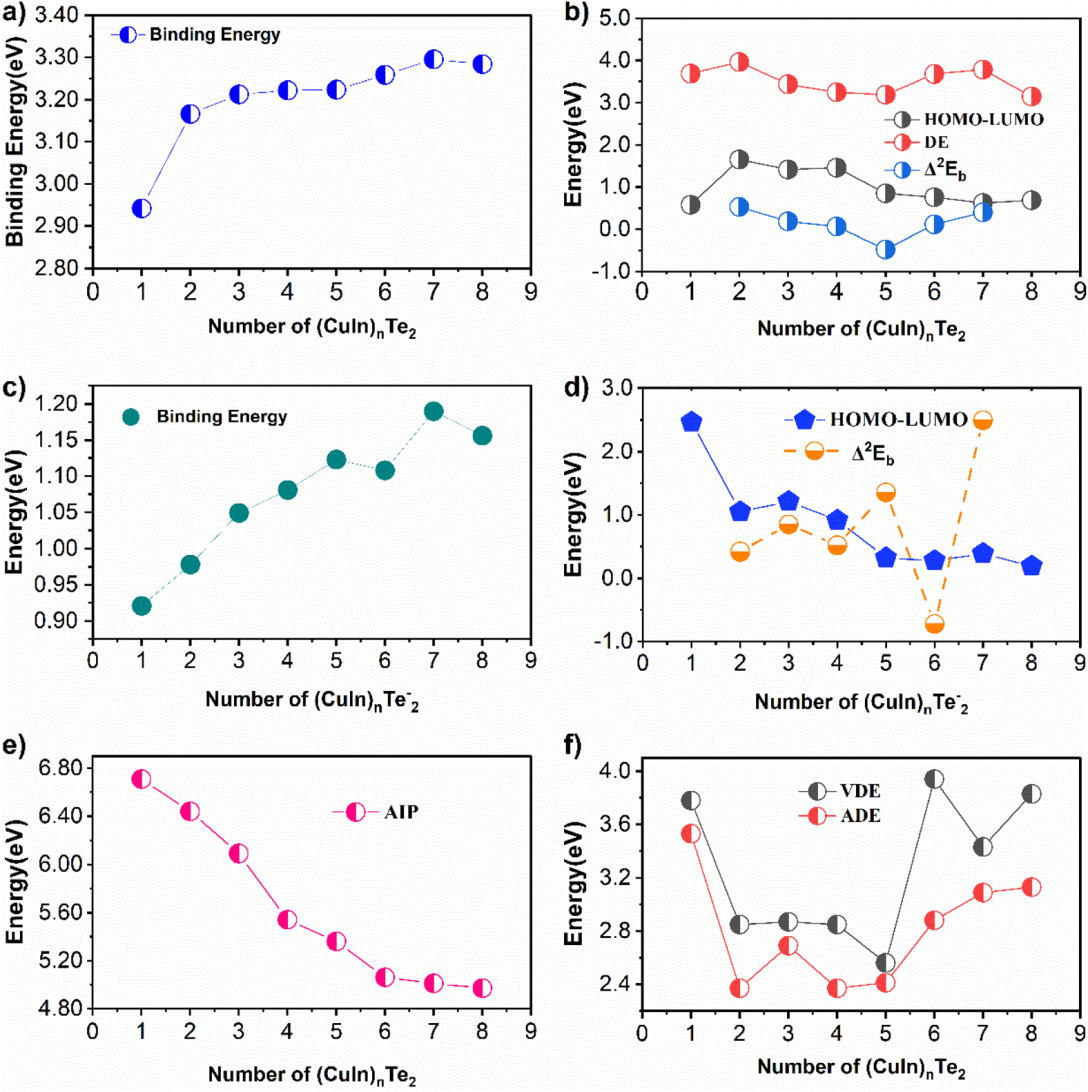
Binding energy (a) comparison of HOMO–LUMO, DE and Δ^2^*E*_b_ (b) of (CuIn)_*n*_Te_2_, Binding energy (c) comparison of HOMO–LUMO, and Δ^2^*E*_b_ (d) of ((CuIn)_*n*_Te_2_)^−^, AIP (e) and VDE and ADE (f) for *n* = 1–8 clusters.

For (CuIn)_3_Te_2_ clusters, an energy range of 0.135 eV (Fig. 1(d), 3(a–d)) show the existence of all four isomers are obtained by adding a CuIn unit to the minimum-energy structure of the Cu_2_In_2_Te_2_ cluster is useful to obtain the lowest-energy structure. For (CuIn)_3_Te_2_ cluster, all four isomers are within an energy range of 0.135 eV (Fig. 1(d), 3(a–d)). The lowest energy structure is obtained by adding CuIn unit to the minimum energy structure of Cu_2_In_2_Te_2_ cluster. Isomer 3(a) is a cubic structure with a composite of one triangular prism and one square pyramid folded with one triangular pyramid. This compound possesses five Cu–In bonds and two Cu–Cu bonds. In the neutral state, the average bond lengths are 2.63 Å and 2.45 Å respectively, while in the anionic state they are 2.48 Å and 2.31 Å respectively. Furthermore, in the neutral state, the average bond angles between adjacent Cu–In–Cu and Cu–Te–In are 54.59° and 71.67°, respectively. Isomer 3(b) adopts a cubic structure, specifically a pentagonal pyramid form where two parallel indium atoms bond to a single copper atom. Isomer 3(c) evolved from isomer 2(b), which possesses a planar rhombic structure with two copper atoms and two indium atoms bonded. This is a hexagonal pyramid form where the Cu–In bond is extended from the structural center. Isomer 3(d) is a variant of isomer 2(b), exhibiting a triangular prism structure with one indium atom positioned on one face and one tellurium atom on the opposite face. The most stable structure 3(a), characterized by strong Cu–Cu bonds converging towards the center, possesses energy 0.104 eV lower than isomer 3(b). This structure exhibits a HOMO–LUMO gap of 1.414 eV and bond energy of 3.213 eV. The ADE/VDE calculations for all isomers yielded values of 2.69/2.87 eV, 2.97/2.59 eV, and 2.90/2.57 eV respectively, in ascending order of energy. When the (CuIn)_3_Te_2_ cluster gains an electron to become an anion, the added negative charge strengthens the attraction between the positively charged copper (Cu) atoms and the negatively charged tellurium atoms. This causes the atoms to move closer together, shortening the bond length. The additional electron introduced into the anion can alter the electron distribution around the atoms. This affects the orbital overlap between Cu and Te, opening the possibility of forming a more stable structure at the shorter bond length. In some cases, electron acquisition can induce structural changes within the molecule. ((CuIn)_3_Te_2_)^−^ adopt a different structure compared to the neutral molecule, potentially gaining stability through the shortening of specific bonds.

#### 3.2.4. (CuIn)_4_Te_2_/((CuIn)_4_Te_2_)^−^ clusters

The lowest-energy structure of the neutral (CuIn)_4_Te_2_ cluster is established by adding CuIn units to all three isomers of the neutral (CuIn)_3_Te_2_ cluster (see Fig. 1(d)). The Cu–Cu and Cu–In bond lengths fall within the ranges of 2.36 Å to 2.42 Å and 2.48 Å to 2.61 Å, respectively. Consequently, the structure of isomer 4(a) is pentagonal pyramidal with a rhombic extension connected to a distorted triangular prism. The Cu–In–Cu and Cu–Te–Cu bond angles were measured as 54.4° and 61.3°, respectively. The neutral isomer 4(b) is a distorted cubic structure with a rhombohedral extension. Isomer 4(a) is the most stable structure, possessing an energy 0.099 eV lower than isomer 4(b) and 0.124 eV lower than isomer 4(c). The bond energy is 3.222 eV, and the HOMO–LUMO gap is 1.457 eV.

For the anion counterpart (Fig. 2(b)), three consecutive isomers exist with different geometric orientations and bond lengths compared to the neutral ion. For example, isomer 4(a) has a Cu–Cu bond length between 2.32 Å and 2.38 Å, and a Cu–In bond length between 2.49 Å and 3.03 Å. Additionally, two further Cu–In bonds with a bond length of 2.73 Å are present. Within these clusters, the distribution of positive and negative charges differs from that in neutral clusters. This charge distribution can enhance electrostatic interactions between ions, potentially promoting the formation of additional bonds. Instead of traditional electron sharing, the dominant electron pair fluctuation characterizes charge transfer (CT) bonding. This bond itself is relatively strong, but it is accompanied by strong covalent-ionic resonance interactions, reduced charge density between the bonded atoms, and properties typically associated with repulsive interactions. As ions reconfigure to reduce electrostatic potential energy, this charge redistribution can facilitate the formation of new bonds.^35^ These changes enable additional bonds to form as ions rearrange to minimize electrostatic potential energy. Therefore, the generation of additional Cu–In bonds in anionic clusters compared to neutral clusters can be attributed to differences in charge distribution, bonding preferences, and structural stability requirements. The calculated ADE/VDE values for each isomer are 2.37/2.85, 2.43/3.39, and 2.87/2.57 eV, respectively.

#### 3.2.5. (CuIn)_5_Te_2_/((CuIn)_5_Te_2_)^−^ clusters

Our simulations generated four minimum-energy isomers within an energy range of 0.135 eV for the neutral (CuIn)_5_Te_2_ cluster. The lowest-energy structure in the neutral cluster is a triple-capped non-planar orthogonal arrangement, with a copper atom positioned at the center. This is clearly evident in Fig. 2(c), 5(a–d), which display the highest degree of cooperation. The Cu–Cu bond lengths for this isomer range from 2.44 Å to 2.60 Å, while the Cu–In bond lengths range from 2.48 Å to 2.91 Å. Due to a negligible energy difference of 0.005 eV, the first two isomers, 5(a) and 5(b), and the subsequent two isomers, 5(c) and 5(d), are energetically almost degenerate. The Cu–In–Cu and Cu–Te–Cu bond angles for isomer 5(a) are 56.7° and 61.7°, respectively. Isomer 5(b) is a distorted double square pyramid complex formed by the bonding of two square pyramids, consisting of one triangular prism composed of symmetrical copper atoms and a distorted triangular pyramid growing from the pyramid's side. Isomer 5(c) possesses a structure where a square pyramid is bonded to a triangular prism, featuring an extension of a pentagonal pyramid where tellurium and indium atoms have grown on the opposite faces of the pyramid. The most stable structure is 5(a), where strong covalent bonds between Cu atoms converge towards the center. This is supported by the highest bond energy of 3.224 eV and a uniform LUMO gap of 0.852 eV.

The bond lengths and angles in the anion region differ from those of the neutral molecule, as can be seen in Fig. 2(d). The fact that the average bond length of the ((CuIn)_5_Te_2_)^−^ dimer is somewhat longer than that of the neutral molecule clearly indicates a weakening of the bonds within the dimer. This is because the force binding the atoms decreases as the bond length increases. The Cu–In, Cu–Te, Cu–Cu, and In–In bond lengths fall within the ranges of 2.52–2.57 Å, 2.54–2.67 Å, 2.37–2.64 Å, and 3.08–3.13 Å respectively. When the bond length of an anion is significantly longer than that of its neutral form, interactions between anions may influence the bond distance. This can arise from changes in anion size, charge effects, or differences in lattice structure. Interestingly, the Cu–Cu interaction is largely preserved, as in the previous neutral state. The calculated ADE/VDE values for each isomer are 2.41/2.56 eV, 2.50/2.67 eV, and 2.16/2.59 eV respectively. Thus, the extended bond lengths, persistent Cu–Cu interactions, and moderate to high ADE/VDE values suggest that electron attachment stabilizes the cluster without disrupting the core metal–metal bond. The differences between isomers highlight the subtleties of geometric and electronic fluctuations governing ionic stability and electron bond strength, demonstrating that relaxation effects play a pivotal role in the dissociation process.

#### 3.2.6. (CuIn)_6_Te_2_/((CuIn)_6_Te_2_)^−^ clusters

The minimum energy geometry of the (CuIn)_6_Te_2_ cluster exhibits three isomers, all of which are considered to represent three-dimensional structures. As seen in Fig. S1, isomer 6(a) consists of two triangular bipyramids, one distorted square pyramid, and one triangular prism. An additional tellurium atom extends from the opposite side of the structure. This structure possesses five double-bridged planar rhombuses formed by two copper atoms and two indium atoms, and four double-bridged planar rhombuses formed by copper atoms. Owing to these common structural motifs, isomer 6(a) can be identified as an isomeric form of structure 5(b) depicted in Fig. 2(c). Cu–In bonds are the most numerous with an average bond length of 2.63 Å (14 bonds), while Cu–Cu bonds are the second most numerous with an average bond length of 2.48 Å (8 bonds). The average bond angles for Cu–In–Cu and Cu–Te–Cu are calculated as 57.1° and 54.3°, respectively. Isomer 6(b) can be viewed as a distorted hexagon with a triangular upper part and a distorted rhombic lower part, where the tellurium atom extends from the right side of the structure, forming a structure resembling an almost spherical icosahedron. Isomer 6(c), formed by the growth of isomer 5(b), consists of a rectangular pyramid superimposed upon the distorted hexagon, along with two pentagonal pyramids bonded to the base and front faces of the structure. Isomer 6(a) is the most stable structure, being 0.047 eV lower than 6(b) and 0.127 eV lower than 6(c). Its HOMO–LUMO gap energy is 0.759 eV, and its binding energy is 3.259 eV. The bond lengths and bond angles of the anionic isomer (see Fig. S1(b)) differ markedly from those in the neutral state. For the anionic isomer (see Fig. S1(b), 6(a)), the average bond lengths for Cu–In, Cu–Te, and Cu–Cu are 2.73 Å, 2.56 Å, and 2.53 Å respectively, with the centrally located copper atom possessing the highest coordination number. This geometric structure comprises a modified triangular prism featuring a copper cap with tellurium at the front and indium at the top. Furthermore, a tetrahedral structure has been modified and integrated within the dihedral structure. In order of increasing energy difference, the calculated ADE/VDE values for the isomers are 2.88/3.94, 3.00/3.79, and 3.12/3.52 eV respectively.

#### 3.2.7. (CuIn)_7_Te_2_/((CuIn)_7_Te_2_)^−^ clusters

Increasing the number of Cu–In units to seven results in the formation of a double-bridged planar arrangement of copper atoms in (CuIn)_6_Te_2_, analogous to that observed in the (CuIn)_7_Te_2_ cluster. These calculations confirm the existence of three stable isomers within this particular cluster. The lowest-energy structure of (CuIn)_7_Te_2_ corresponds to a more symmetrical copper atom arrangement (Fig. S1(c), 7(a)). Here, the single copper atom at the center forms the strongest bonds with its nearest neighbors. Isomer 7(a) possesses a distorted pentagonal bipyramid cap structure with triangular pyramids positioned at the top and bottom. The average bond lengths are 2.47 Å for Cu–Cu and 2.69 Å for Cu–In. The characteristic bond angles are 56.08° for Cu–In–Cu and 71.89° for Cu–Te–Cu, reflecting the typical geometric arrangement of the structure. This structure is a variant of isomer 2(b), possessing a distorted small spherical structure with four double-bridged planar rhombic structures. Isomer 7(b) is a distorted small spherical structure with a single double-bridged planar rhombic structure. The copper atom is located at the center of the entire structure and bonds to seven atoms. Isomer 7(c) is also a complex structure where a distorted pentagon is covered by a square pyramid, with two triangular prisms bonded to its base. This geometric structure comprises ten ionic Cu–Te and In–Te bonds and thirty covalent Cu–In interactions. The most stable structure is 7(a), which is 0.132 eV lower than isomer 7(b) and 0.443 eV lower than isomer 7(c), with a bond energy and HOMO–LUMO gap of 3.295 eV and 0.619 eV respectively.

In the anion counterparts with opposite charges (see Fig. S2(d) and 7(a)), the average bond lengths for Cu–In, Cu–Te, and Cu–Cu are 2.62 Å, 2.50 Å, and 2.43 Å respectively, with the centrally located copper atom possessing the highest coordination number. The highest binding energy belongs to this cluster. This is therefore expected to arise from the complete coordination of the copper atom (or the absence of dangling bonds and a highly symmetrical structure). Due to indium's relatively large atomic radius, the In–In bond exhibits the longest bond length in both neutral and anionic species. In order of increasing energy, the calculated ADE/VDE values for the isomers are 3.09/3.43 eV, 2.94/3.51 eV, and 3.16/2.83 eV, respectively. For the first and second isomers, the VDE value exceeds the ADE value, suggesting significant structural relaxation due to electron separation and the formation of a distinct anionic minimum energy state. The second isomer exhibits the highest VDE (3.51 eV), suggesting the strongest vertical electron bonding. Conversely, the third isomer shows a smaller VDE than ADE, indicating reduced vertical stability and the potential for structural sensitivity during electron removal. Overall, the ADE/VDE trend supports the conclusion that while all isomers are stable in the anionic state, distinct electronic bonding strengths and relaxation behaviors arise due to differences in geometric and electronic structures.

#### 3.2.8. (CuIn)_8_Te_2_/((CuIn)_8_Te_2_)^−^ clusters

Adding Cu–In dimer units to the two isomers of the neutral (CuIn)_7_Te_2_ cluster yields the two isomers of the neutral (CuIn)_8_Te_2_ cluster shown in Fig. S1(e), 8(a) and (b). Isomer 8(a) features five stacked distorted triangular prisms, each topped by two distorted square pyramids. This structure was confirmed as the most stable, exhibiting the lowest-energy arrangement with average bond lengths of Cu–Cu 2.49 Å, Cu–In 2.70 Å, and Cu–Te 2.52 Å. The average bond length for In–In is 3.04 Å. The bond lengths for each pair harmonize with those in the bulk structure. Isomer 8(b) features a folded twisted cubic structure, with twisted square pyramids covering the left and top faces. Additionally, the number of tellurium atoms near the structural vertices has increased. Copper is positioned closer to the center, forming eight bonds with its nearest neighbors, resulting in energy 0.047 eV higher than isomer 8(a). The HOMO–LUMO gap is 0.682 eV and the bond energy is 3.284 eV. In the anionic structure (Fig. S1(f), 8(a)), the typical bond lengths for Cu–Cu, Cu–In, and Cu–Te are 2.46 Å, 2.59 Å, and 2.51 Å respectively, with the centrally located copper atom possessing the largest coordination number. Furthermore, the average In–In bond length is 3.04 Å, identical to the value for the neutral cluster. The ADE/VDE values for each isomer, calculated in order of increasing energy, are 3.13/3.81 eV and 3.05/5.41 eV respectively.

### 3.3. Electronic properties of (CuIn)_*n*_Te_2_/((CuIn)_*n*_Te_2_)^−^ clusters

To assess the stability of the clusters, the binding energy and HOMO–LUMO gap were investigated. As shown in Fig. 3(a), the binding energy generally increases with the number of CuIn units, though the rate of increase slows as the cluster grows larger. Overall, the (CuIn)_*n*_Te_2_ clusters exhibit high binding energies, though their values show some variability. Therefore, this cluster exhibits remarkable stability. The binding energy varies depending on the coordination number and symmetry of the cluster. The nearly saturated binding energy beyond *n* = 2 may be due to the structural similarity between larger (CuIn)_*n*_Te_2_ clusters, and possibly the presence of a cubic Cu_2_In_2_Te_2_ motif. Neutral clusters starting from *n* = 2–5 exhibit a pattern of nearly gradual changes in binding energy compared to the *n* = 1 cluster. However, Cu_7_In_7_Te_2_ displays a larger binding energy than the other surrounding clusters. The average Cu–Te bond length in Cu_7_In_7_Te_2_ is very short at 2.47 Å. Consequently, the high binding energy of these clusters stems from the strong bonding between Cu and Te atoms within the cluster. As seen in Table 1, the calculated average In–Te and In–In bond lengths are generally larger than the average Cu–Te and Cu–In bond lengths within the cluster. This is an expected result, as the atomic radii of copper and tellurium are smaller than those of indium and tellurium. This also agrees with the experimental bond length result of 2.76 Å.^36^ The energy difference between the highest occupied molecular orbital (HOMO) and the lowest unoccupied molecular orbital (LUMO) serves as a measure of cluster stability, with a larger HOMO–LUMO (HL) gap indicating enhanced chemical stability.

**Table 1:** Energetic properties (eV) of first isomers (CuIn)_*n*_Te_2_ for *n* = 1–8 neutral clusters

(CuIn)_*n*_Te_2_	BE	H–L gaps	DE	Δ^2^*E*_b_	VDE	ADE	AIP	Ref.
CuInTe_2_	2.942	0.577	3.685		3.78	3.53	6.71	5
Cu_2_In_2_Te_2_	3.166	1.652	3.958	0.53	2.85	2.37	6.44	This work
Cu_3_In_3_Te_2_	3.213	1.414	3.436	0.18	2.87	2.69	6.09	
Cu_4_In_4_Te_2_	3.222	1.457	3.252	0.06	2.85	2.37	5.54	
Cu_5_In_5_Te_2_	3.224	0.852	3.194	−0.48	2.56	2.41	5.36	
Cu_6_In_6_Te_2_	3.259	0.759	3.678	0.11	3.94	2.88	5.06	
Cu_7_In_7_Te_2_	3.295	0.619	3.782	0.40	3.43	3.09	5.01	
Cu_8_In_8_Te_2_	3.284	0.682	3.149		3.83	3.13	4.97	

This is used to describe the extent to which a molecule can participate in chemical reactions. The larger the HOMO–LUMO gap value, the more energy is required for an electron to transition from the HOMO level to the LUMO level. Through graphical analysis, the size dependence of each cluster is discussed as follows. Generally, the HOMO–LUMO gap of (CuIn)_*n*_Te_2_ clusters varies with size, exhibiting a decreasing trend as the size increases. For example, the HOMO–LUMO gap for (CuIn)_2_Te_2_ is 1.65 eV, whereas for CuInTe_2_ it is 0.58 eV.

Experimental studies on I–III–VI_2_ ternary semiconductor compounds manufactured *via* spray pyrolysis revealed a variable direct bandgap ranging from 0.92 to 1.89 eV.^14,37^ Calculated HOMO–LUMO results exhibited odd–even oscillations, showing a significant decrease starting from (*n* = 5), whereas the HL gap remained nearly unchanged from *n* = 5 to *n* = 8.

Furthermore, clusters characterized by copper–copper atomic contacts within the core throughout the optimization process were found to exhibit relatively small HOMO–LUMO gaps between isomers. The number of coordination sites occupied by each copper and tellurium atom within the cluster also influences the HL gap, as shown in Fig. S1c. Clusters exhibiting favorable atomic coordination generally exhibit the lowest HL gap.

To investigate relative stability, it is more useful to analyze the second-order energy difference between a cluster and its neighboring clusters. Fig. 3(b) depicts the graph of the secondary energy difference Δ^2^*E*_b_(*n*) for (CuIn)_*n*_Te_2_ clusters per CuIn unit. These clusters exhibit a distinct odd–even vibration pattern, although the odd–even vibrations are somewhat hindered by geometric factors.

The greater the value of Δ^2^*E*_b_(*n*) (the more positive it is), the higher the stability; negative values indicate low stability. This signifies that monomer exchange between homologous pairs is favorable. As seen in Fig. 3(b), the peak corresponding to *n* = 2 can be classified as a ‘magic number’ cluster, suggesting this cluster is more stable than neighboring clusters. It is noteworthy that among clusters of all sizes, the (CuIn)_2_Te_2_ cluster exhibits the strongest relative stability in terms of the calculated cluster energy Δ^2^*E*_b_(*n*). This is strongly supported by the HOMO–LUMO gap and molecular energy (DE) shown in Fig. 3(b). The large HOMO–LUMO difference within the cluster may be attributed to the closed 8-electron shell and the highly symmetrical geometric structure. The DE curve exhibits peaks at *n* = 2 and 7. These peaks indicate that higher energy is required to liberate the CuIn unit from the (CuIn)_*n*_Te_2_ cluster. Therefore, Cu_2_In_2_Te_2_ can be considered a relatively more prominent cluster, suggesting a chemically stable state.

Table 2 provides an overview of the energetic characteristics of the anion counterparts. As can be seen in Fig. 3(c), the binding energy of the clusters increases monotonically with cluster size. It increases approximately linearly with cluster size up to *n* = 5, after which size-dependent oscillations begin. At *n* = 6, a sharp positive peak is observed, indicating the maximum binding energy relative to the surrounding clusters. The HOMO–LUMO gap shows a clear alternating pattern of odd and even values, ranging from 0.194 to 2.464 eV. It shows a significant decrease at *n* = 3, followed by only small fluctuations at *n* = 5 to 8. In general, clusters with larger openings are more stable and less reactive. It is noteworthy that the (CuInTe_2_)^−^ cluster exhibits the largest HOMO–LUMO gap of all the anionic clusters investigated. As can be seen in Fig. 3(d), Δ^2^*E*_b_(*n*) oscillates with cluster size and reaches a maximum at *n* = 3, 5 and 7, indicating increased stability for these clusters. In particular, the (Cu_7_In_7_Te_2_)^−^ cluster exhibits the highest Δ^2^*E*_b_(*n*) value, making this cluster the most stable of all sizes investigated. This exceptional stability is likely related to the highly symmetrical structure with a central copper atom.

**Table 2:** Energetic properties (eV) of the first anion isomer of ((CuIn)_*n*_Te_2_)^−^ for *n* = 1–8 clusters

((CuIn)_*n*_Te_2_)^−^	BE	H–L gaps	Δ^2^*E*_b_	Ref.
(CuInTe_2_)^−^	0.921	2.464		5
(Cu_2_In_2_Te_2_)^−^	0.978	1.048	−0.330	This work
(Cu_3_In_3_Te_2_)^−^	1.049	1.212	0.097	
(Cu_4_In_4_Te_2_)^−^	1.081	0.916	−0.233	
(Cu_5_In_5_Te_2_)^−^	1.123	0.323	0.605	
(Cu_6_In_6_Te_2_)^−^	1.108	0.278	−1.474	
(Cu_7_In_7_Te_2_)^−^	1.190	0.389	1.746	
(Cu_8_In_8_Te_2_)^−^	1.156	0.194		

Ionization potential and electron affinity are two other ways of assessing relative stability. Atoms with lower ionization energy easily lose their valence electrons and become cations, which can form anion bonds. Smaller electron affinity values for copper–indium telluride clusters often indicate greater stability compared to nearby clusters. The VDE and ADE values and the ADE-based estimates of the electron affinities of the respective neutral clusters are plotted against the number of CuIn units in Fig. 3(e) and (f). Clusters with an even number of units consistently show lower values than clusters with an odd number, according to the alternating trend in the data.

These values increase monotonically from *n* = 5 onwards, but for instance, the ADE for the Cu_7_In_7_Te_2_ cluster is 2.41 eV, whereas that for the (CuIn)_8_Te_2_ cluster is 3.13 eV. For relatively small clusters (*n* = 2–4), the VDE and ADE values are nearly identical to those of neighboring clusters. This clearly indicates a reluctance to accept charge units and a preference for non-metallic tendencies. A larger HOMO–LUMO gap correlates with smaller vertical and adiabatic ionization energies, and *vice versa*. To gain fundamental insights into the electronic structure and chemical reactivity of (CuIn)_*n*_Te_2_ clusters (*n* = 1–8), the AIP of the clusters shown in Fig. 3(e) was calculated. As can be seen in the figure, the AIP value decreases monotonically from *n* = 1 (6.71 eV) to *n* = 8 (4.97 eV). Generally, the AIP value decreases as the cluster size increases. Clusters with large AIP values tend to exhibit non-metallic characteristics and demonstrate stability in participating in chemical reactions.

### 3.4. Partial charge density analysis of clusters

The density of states (DOS) provides an intuitive method for characterizing complex electronic structures and represents one of the most fundamental concepts for interpreting a material's physical properties. Band gaps and effective masses are two key elements visually identifiable within the DOS, forming the basis for a material's electrical and optical characteristics. Calculating the density of atomic states (DOS) with high quality to accurately represent a material's electronic structure greatly facilitates the interpretation and adjustment of various material properties.^38,39^ A high DOS at a specific energy level (*E* to *E* + d*E*) indicates a large number of available states, whereas a DOS of zero signifies no available states at that energy. The entire DOS can be decomposed into the contributions of atomic orbitals (s, p, d).^5^ For micro clusters, quantum confinement effects dominate in all three dimensions, forming discrete energy levels instead of continuous energy bands. Consequently, the DOS appears as a series of sharp peaks, similar to the discrete energy levels of molecules and atoms, unlike the continuous energy bands observed in bulk materials.^40^

#### 3.4.1. Partial charge density analysis (CuIn)_*n*_Te_2_ cluster

This enables the explanation of charge delocalization within the cluster, the characteristics of individual molecular orbitals, and the phenomenon of molecular orbital overlap.^21,41^ For the purposes of discussion, the most stable Cu_2_In_2_Te_2_ cluster was selected as the representative cluster. As shown in Table S2, the partial charge density was calculated for some occupied and unoccupied orbitals around the Fermi level of the (CuIn)_*n*_Te_2_ cluster.

At the HOMO level, the molecular orbital receives a significant contribution from the d orbitals of the Cu atom, a negligible contribution from the p orbitals of the Te atom, and no contribution whatsoever from the s orbitals. This indicates that the HOMO is primarily localized on the Cu atom, as shown in Fig. S2(a). Similarly, the HOMO−1 and HOMO−2 levels originate mainly from the d orbitals of Cu, while the contribution of the p orbitals of Te atoms is negligible. No contribution from the s orbitals of In atoms is observed. Consequently, HOMO−1 remains mainly confined to the Cu and Te atoms. The contribution to the LUMO orbital mainly originates from the p orbitals of Te and the s orbitals of the In atom.

Minimal contribution from the d orbital of the Cu atom. For the LUMO+1 level, the most significant contributions come from the s orbitals of Cu and the p orbitals of Te atoms. Fig. S2 (a) shows that the most significant contribution to the LUMO+2 orbital comes from the p orbital of both In and Te atoms. A small contribution from the d orbitals of the Cu atom is also shown. An inspection of the LDOS in Fig. S2(b), combined with the charge density analysis in Table S2, reveals that in the vicinity of the Fermi level, the dominant contribution comes from Cu atoms. The energy level diagram in Fig. S2(b) shows that the CuInTe_2_ cluster has a Fermi level that lies below the mid-gap, *i.e.* close to the HOMO level. However, the difference is negligible, resulting in a band shift in energy towards the higher energy levels. The partial charge density distributions of the HOMO−1, HOMO, LUMO, and LUMO+1 orbitals for Cu_2_In_2_Te_2_ and Cu_2_In_2_Te_2_^−^ clusters are shown in Table 3. The p orbital of Te and the d orbital of the Cu atom are where the HOMO is almost completely concentrated for both clusters. Both clusters also show a small contribution from the s and p orbitals of an atom. The p orbitals of the indium (In) atom and the d orbitals of the copper (Cu) atom possess strongly localized LUMO levels in both neutral and anionic clusters. The s and d orbitals of the copper atom and the s orbital of the phosphorus atom are the primary contributors to the LUMO+1 level in the neutral cluster. Additionally, a minor contribution exists from the p orbital of the terbium atom.

**Table 3:** Partial charge density of state within Cu, In, and Te atoms calculated for some occupied and unoccupied orbitals in Cu_2_In_2_Te_2_ (A) and (Cu_2_In_2_Te_2_)^−^ (B) clusters

A					B				
Orbital	Atom	s	p	d	Orbital	Atom	s	p	d
HOMO−1	Cu	0.005	0.001	0.032	HOMO−1	Cu	0.000	0.006	0.034
	Cu	0.001	0.004	0.152		Cu	0.002	0.007	0.099
	In	0.029	0.017	0.000		In	0.010	0.018	0.001
	In	0.000	0.027	0.003		In	0.023	0.035	0.002
	Te	0.000	0.253	0.000		Te	0.000	0.101	0.000
	Te	0.000	0.012	0.000		Te	0.000	0.116	0.000
HOMO	Cu	0.002	0.003	0.072	HOMO	Cu	0.000	0.007	0.050
	Cu	0.000	0.003	0.046		Cu	0.001	0.006	0.028
	In	0.031	0.020	0.002		In	0.032	0.022	0.000
	In	0.006	0.004	0.000		In	0.005	0.013	0.001
	Te	0.000	0.013	0.000		Te	0.000	0.038	0.000
	Te	0.006	0.248	0.000		Te	0.000	0.193	0.000
LUMO	Cu	0.010	0.024	0.135	LUMO	Cu	0.002	0.001	0.138
	Cu	0.001	0.005	0.129		Cu	0.057	0.021	0.112
	In	0.003	0.097	0.002		In	0.005	0.165	0.001
	In	0.024	0.130	0.002		In	0.005	0.179	0.003
	Te	0.002	0.038	0.001		Te	0.001	0.016	0.000
	Te	0.000	0.059	0.000		Te	0.000	0.048	0.000
LUMO+1	Cu	0.126	0.028	0.120	LUMO+1	Cu	0.002	0.012	0.125
	Cu	0.112	0.005	0.125		Cu	0.011	0.020	0.132
	In	0.132	0.047	0.000		In	0.113	0.051	0.001
	In	0.007	0.105	0.004		In	0.013	0.026	0.000
	Te	0.001	0.115	0.002		Te	0.001	0.066	0.002
	Te	0.002	0.027	0.001		Te	0.001	0.098	0.001

**Fig. 4:**
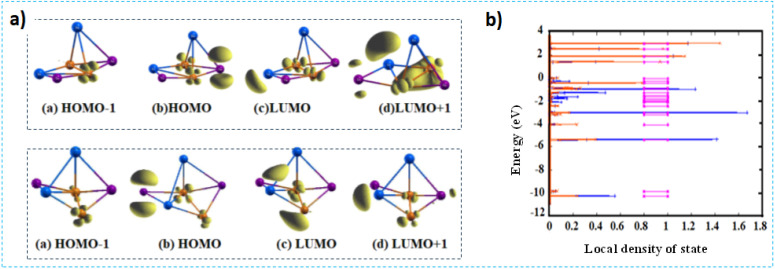
Partial charge density plots for the HOMO, LUMO, and LUMO+1 orbitals of the Cu_2_In_2_Te_2_ cluster in neutral and anionic states (a). Contour plots are displayed at 1/5^th^ of the maximum value. LDOS and energy levels of Cu_2_In_2_Te_2_ (orange) and Cu_2_In_2_Te_2_^−^ clusters. A Gaussian function with a width of 0.01 eV broadens the discrete spectrum (b).

For the anion, the LUMO+1 level is confined to the d orbitals of the Cu atom and the s orbitals of the In atom. Fig. 4 shows the minimal contribution from the p orbitals of the terbium atom, consistent with the results summarized in Table 3. Table 3 as shown in the table, both the HOMO−1 and HOMO levels are scarcely affected by the atomic s orbitals near the Fermi level in both the neutral and anionic states. The indium atom is an exception. In both neutral and anionic clusters, the distribution of HOMO and LUMO orbitals is primarily concentrated on the Cu and Te atoms respectively. A comprehensive review of the LDOS plot (Fig. 4(b)) and the charge density analysis presented in Table 3 reveals that the major contribution near the Fermi level originates from the Te atoms. In the energy diagram (Fig. 4(b)), the Fermi level of the Cu_2_In_2_Te_2_ cluster is positioned below the mid-bandgap and is closest to the HOMO level. However, a slight shift towards higher energies is observed due to band displacement. Consequently, the 5p electrons of the Te and In atoms play a pivotal role in the interactions between the In and Te atoms.

## 4. Conclusion

Herein, we investigated the electronic and structural properties of (CuIn)_*n*_Te_2_ (*n* = 1–8) and their anionic counterparts using first-principles DFT. The optimized structures show that Cu atoms preferentially occupy central, highly coordinated positions, and the most stable neutral clusters, especially Cu_2_In_2_Te_2_ and Cu_4_In_4_Te_2_ exhibit large HOMO–LUMO gaps and enhanced chemical stability, with slight odd–even oscillations arising from electron-pair effects. In contrast, the anionic clusters are more stable for odd-sized species. Both neutral and anionic clusters generally possess sizable HOMO–LUMO gaps (0.524–2.464 eV), indicating chemical inertness and tunability of electronic properties through cluster size. Trends in ADE, VDE, and ionization potentials further reveal increasing stability and a gradual shift toward metallic behavior with increasing cluster size. These results highlight stable Cu–In–Te building units, support the potential of I–III–VI_2_ chalcopyrite systems for bandgap engineering, and provide a foundation for future studies on self-assembled clusters and applications in thin-film solar cells, semiconductors, and energy-storage materials.

## Author contributions

Kidane Goitom Gerezgiher: writing, visualization, methodology, software, investigation, data curation, conceptualization and editing. Bereket Woldegbreal Taklu: visualization, reviewing, editing. Taame Abraha Berhe: validation, editing, reviewing, Teklay Mezgebe Hagos, editing and reviewing and Hagos Woldeghebriel Zeweldi participated in supervising, technical matters, reviewing, resources, conceptualization, and editing of the manuscript. All authors agree with the final form of the manuscript.

## Conflicts of interest

The authors declare no competing financial interest.

## Supplementary Material

RA-016-D6RA00246C-s001

## Data Availability

Additional details or computational files can be provided by the corresponding author upon reasonable request. Additional data and computational details are provided in the supplementary information (SI). Supplementary information is available. See DOI: https://doi.org/10.1039/d6ra00246c.
