# Supplementary material for: Structural evolution and stabilities of (CuIn)nTe2 and ((CuIn)nTe2)− (n = 1–8) clusters via DFT study
Source: RSC Adv. 2026 Jan 28;16(7):6106–18. doi: 10.1039/d6ra00246c (PMC12851157; doi:10.1039/d6ra00246c)
Supplement: RA-016-D6RA00246C-s001 [file RA-016-D6RA00246C-s001.pdf]

Supplementary Information (SI) for RSC Advances.
This journal is © The Royal Society of Chemistry 2026

Supporting Information

Structural Evolution and Stabilities of $(CuIn)_nTe_2$ and $((CuIn)_nTe_2)^-$ (n = 1 - 8) Clusters via DFT Study

Kidane Goitom Gerezgiher, [1]* Bereket Woldegbreal Taklu,[2] Taame Abraha Berhe,[3] Teklay Mezgebe Hagos,[3] Hagos Woldeghebriel Zeweldi [4]*

[1]Department of Physics, Abbiyi Addi College of Teachers Education and Educational Leadership, Abbiyi Addi, P.O.Box 11, Ethiopia,

[2]Department of Chemistry, CNCS, P.O. Box 1145, Wollo University, Dessie, Ethiopia.

[3]Department of Chemistry, CNCS, P.O. Box 50, Adigrat University, Adigrat, Ethiopia.

[4]Department of Physics, CNCS, P.O. Box 231, Mekelle University, Mekelle, Ethiopia.

Corresponding authors

Kidane Goitom Gerezgiher; kidanegoitom5@gmail.com,

Hagos Woldeghebriel; hagos93@mu.edu.et

Methodology used for the DFT calculations

The VASP is used to examine the electronic and structural properties of neutral and anionic, $(CuIn)_nTe_2$ clusters[1]. The package uses density functional theory (DFT) with a plane-wave and ultrasoft pseudopotential basis set. The arrangement of atoms inside a supercell that repeats on a regular basis serves as the input for VASP. Atomic mobility and the electrical structure and energy of the atomic configuration are the most basic outputs. If the cluster length is less than 10 Å, we employed a cubic supercell with an edge length of 20 Å simulation box for structure optimization; if not, we used a (30 × 30 × 30) $Å^3$ simulation box with periodic boundary conditions[2]. Furthermore, due to the size of the supercell, only the point (Γ) is used to scan the Brillouin zone. VASP uses a line minimization of the energy along the force direction to determine the minimum. The atoms are relaxed to their present ground state using a conjugate gradient method. Band structure energy is integrated across the entire Brillouin zone using the smearing or tetrahedron method. The density of states was determined using a Gaussian smearing of 0.01 eV. For each system, the plane-wave cutoff energy was set to 240 eV. The structure was considered to have converged when the force acting on each ion, as calculated using the Kohn-Sham energy function, was less than 10^{-4} eV/Å. Quantification of various planar and three-dimensional structures was performed using the conjugate gradient method. The atomic electron configurations and number of electrons considered for each parent molecule were ($3d^{10}4s^1$) for copper (Cu), ($5s^25p^1$) for indium (In) and ($5s^25p^4$) for tellurium (Te). In order to ascertain whether the lowest-energy structure had been discovered, we placed copper, indium and tellurium atoms in multiple hypothetical arrangements, both symmetrical and non-symmetrical.

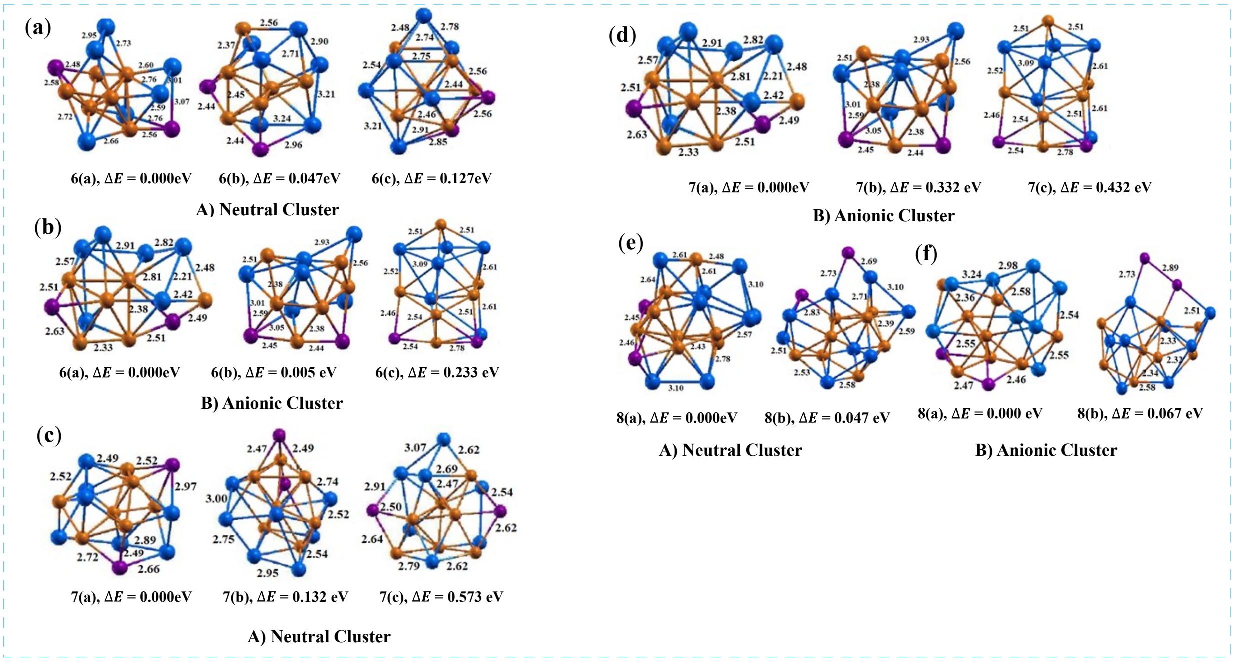

Fig. S1: Fig. S1: Optimized geometry of $(CuIn)_nTe_2$ (a, c, and e) and $((CuIn)_nTe_2)^-$ (b, d, and f) clusters for (n = 6 - 8), with corresponding relative energy ΔE, with respect to the lowest energy structure.

Table S1: Average bond length in (Å) for Cu-In, Cu-Te, In-Te, and In-In, respectively, of the first isomers neutral $(CuIn)_nTe_2$ for n = 1 - 8 clusters.

$(CuIn)_nTe_2$	**Cu-Cu**	**Cu-In**	**Cu-Te**	**In-Te**	**In-In**	**Ref.**
$CuInTe_2$	2.62	2.47	2.51	2.61		[3]
$Cu_2In_2Te_2$	2.65	2.79	2.43	3.02		*This work*
$Cu_3In_3Te_2$	2.45	2.63	2.42	2.63	2.93	
$Cu_4In_4Te_2$	2.40	2.60	2.45	2.93	2.97	
$Cu_5In_5Te_2$	2.52	2.65	2.51	3.03	2.97	
$Cu_6In_6Te_2$	2.48	2.62	2.57	2.92	3.03	
$Cu_7In_7Te_2$	2.47	2.69	2.48	3.05	3.01	
$Cu_8In_8Te_2$	2.49	2.70	3.52	3.01	3.04	

Table S2: *s, p,* and *d* partial charges within Cu, In, and Te atoms calculated for some occupied and unoccupied orbitals in $(CuIn)_nTe_2$ Cluster.

Orbital	Atom	*s*	*p*	*d*	Orbital	Atom	*s*	*p*	*d*
	Cu	0.000	0.025	0.154		Cu	0.002	0.007	0.090
	In	0.000	0.000	0.003		In	0.166	0.025	0.006
	Te	0.000	0.096	0.000	LUMO	Te	0.002	0.186	0.001
**HOMO**	Te	0.000	0.079	0.000		Te	0.002	0.176	0.001
	Cu	0.087	0.029	0.177		Cu	0.176	0.039	0.009
	In	0.000	0.013	0.002		In	0.013	0.009	0.001
	Te	0.001	0.029	0.000	LUMO+1	Te	0.004	0.127	0.005
**HOMO-1**	Te	0.001	0.029	0.000		Te	0.004	0.112	0.005
**HOMO-1**	Cu	0.000	0.007	0.272		Cu	0.000	0.001	0.009
	In	0.000	0.022	0.000		In	0.000	0.173	0.003
	Te	0.000	0.114	0.000	LUMO+2	Te	0.000	0.049	0.005
**HOMO-2**	Te	0.000	0.114	0.000		Te	0.000	0.049	0.005

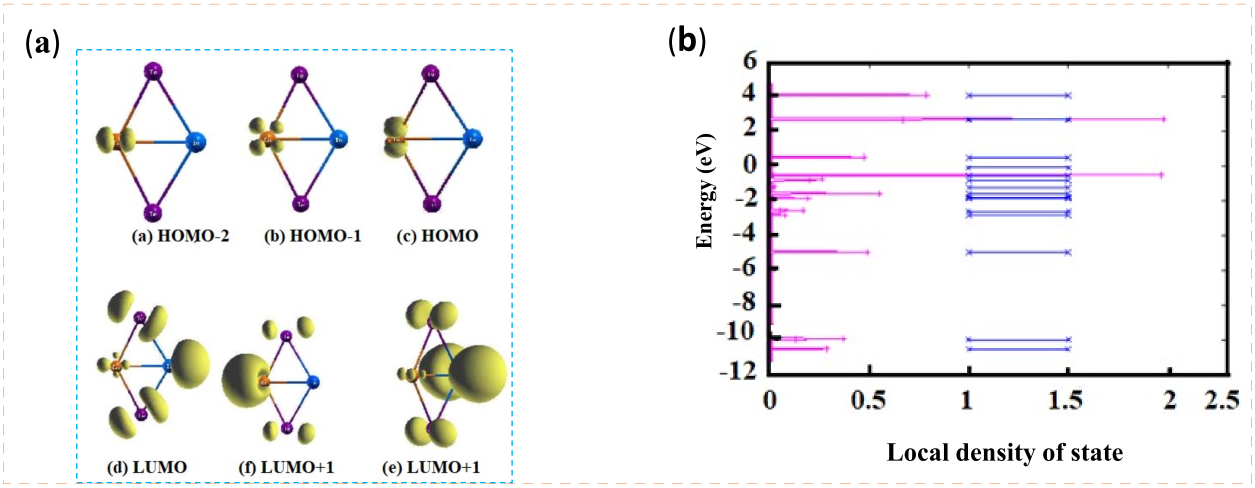

Fig. S2: (**a**) Partial charge density plots of the $(CuIn)_nTe_2$. Subsurface plots are at 1/5th of the maximum value. Red and blue are for Cu and In, respectively. (**b**) LDOS and energy levels of $CuInTe_2$ clusters. The Fermi level is shifted to the zero of the energy axis. The discrete spectra are broadened by a Gaussian of width 0.01eV.

Reference

1. G. Kresse and J. Furthmüller, *Physical review B*, 1996, **54**, 11169.
2. Y. Mulugeta and H. Woldeghebriel, *Computational and Theoretical Chemistry*, 2014, **1039**, 40-49.
3. K. G. Gerezgiher, D. A. Tafere, T. G. Teklehaimanot, A. B. Tadesse, A. M. Dinkirie and H. W. Zeweldi, *RSC advances*, 2025, **15**, 21541-21554.
